# Whole genome sequence revealed the fine transmission map of carbapenem-resistant *Klebsiella pneumonia* isolates within a nosocomial outbreak

**DOI:** 10.1186/s13756-018-0363-8

**Published:** 2018-06-01

**Authors:** Wenjun Sui, Haijian Zhou, Pengcheng Du, Lijun Wang, Tian Qin, Mei Wang, Hongyu Ren, Yanfei Huang, Jing Hou, Chen Chen, Xinxin Lu

**Affiliations:** 1 Department of Clinical Laboratory, Beijing Tongren Hospital, Capital Medical University, Beijing, 100730 China; 2 State Key Laboratory for Infectious Disease Prevention and Control, National Institute for Communicable Disease Control and Prevention, Chinese Center for Disease Control and Prevention, Beijing, 102206 China; 3 Collaborative Innovation Centre for Diagnosis and Treatment of Infectious Diseases, Hangzhou, 310003 China; 4 Beijing Key Laboratory of Emerging Infectious Diseases, Institute of Infectious Diseases, Beijing Ditan Hospital, Capital Medical University, Beijing, 100015 China; 5 Surgical Intensive Care Unit, Beijing Tongren Hospital, Capital Medical University, Beijing, 100730 China

**Keywords:** Carbapenemases, *K. pneumoniae*, KPC-2, Outbreak, Whole genome sequencing

## Abstract

**Background:**

Carbapenem-resistant *Klebsiella pneumoniae* (CRKP) is a major cause of nosocomial infections worldwide. The transmission route of CRKP isolates within an outbreak is rarely described. This study aimed to reveal the molecular characteristics and transmission route of CRKP isolates within an outbreak of nosocomial infection.

**Methods:**

Collecting case information, active screening and targeted environmental monitoring were carried out. The antibiotic susceptibility, drug-resistant genes, molecular subtype and whole genome sequence of CRKP strains were analyzed.

**Results:**

Between October and December 2011, 26 CRKP isolates were collected from eight patients in a surgical intensive care unit and subsequent transfer wards of Beijing Tongren hospital, China. All 26 isolates harbored *bla*_KPC-2_, *bla*_SHV-1_, and *bla*_CTX-M-15_ genes, had the same or similar pulsed-field gel electrophoresis patterns, and belonged to the sequence type 11 (ST11) clone. By comprehensive consideration of genomic and epidemiological information, a putative transmission map was constructed, including identifying one case as an independent event distinct from the other seven cases, and revealing two transmissions starting from the same case.

**Conclusions:**

This study provided the first report confirming an outbreak caused by *K. pneumoniae* ST11 clone co-harboring the *bla*_KPC-2_, *bla*_CTX-M-15_, and *bla*_SHV-1_ genes, and suggested that comprehensive consideration of genomic and epidemiological data can yield a fine transmission map of an outbreak and facilitate the control of nosocomial transmission.

## Background

Carbapenems remain the first-line therapeutic antimicrobials for severe infections caused by extended-spectrum β-lactamase (ESBL)-producing multidrug-resistant *Enterobacteriaceae*. However, the emergence of carbapenemase-mediated resistance to all β-lactams, including carbapenems, is a major public health threat [1, 2]. Over the last decade, carbapenem resistance, attributed to the production of carbapenem-hydrolyzing β-lactamases, has been steadily increasing among *Enterobacteriaceae* isolates, particularly *Klebsiella pneumoniae* [3]. Carbapenem-resistant *K. pneumoniae* (CRKP) has emerged in many countries as a result of intra-continental and inter-continental spread [4–6].

Clinically, the *K. pneumoniae* carbapenemase (KPC) enzyme is one of the most prevalent carbapenemases. The *bla*_KPC_ genes are predominantly plasmid encoded. In some cases, *bla*_KPC_ genes exist in particularly clones (such as *K. pneumoniae* multilocus sequence type (ST) 258 and ST11, which have facilitated their rapid dissemination. Since the first KPC-producing isolate was identified from North Carolina, USA, in 1996 [7], the occurrence of KPC-producing bacteria has been continuously reported in other parts of the USA, Europe, South America, the Middle East, and Asia [5, 8–10]. They have been associated with large nosocomial outbreaks worldwide, including those in China. The CRKP outbreak isolates in China mostly carried *bla*_KPC-2_ [11–14] or *bla*_NDM-1_ [15–18]. KPC-producing members of the family *Enterobacteriaceae* have also been associated with high mortality rates, particularly among critically ill patients with a history of prolonged hospitalization [19–21]. These facts strongly suggest a need for the implementation of adequate preventive measures to effectively control the spread of such pathogens.

From October 8th to December 23th, 2011, a total of 12 CRKP strains were isolated from clinical samples of eight patients admitted to the surgical intensive care unit (SICU). Active screening and targeted environmental monitoring were carried out between October 11th and October 27th, and eight CRKP were isolated from nose and throat of inpatient in SICU, as well as environmental samples. Furthermore, six CRKP were retrieved from one case in subsequent transfer wards. In the present study, we carried out a retrospective investigation of the molecular and genomic epidemiology of the outbreak.

## Methods

### Retrospective analysis of medical records

The medical records of patients from whom CRKP was isolated were reviewed, including time of stay in SICU, bed site, data of the first CRKP isolation, and outcomes. CRKP is defined as resistance to carbapenems according to the Clinical and Laboratory Standards Institute (CLSI) breakpoints [22].

### Active screening and targeted environmental monitoring

Targeting KPC-2 *K. pneumoniae*, active screening and environmental monitoring were carried out as measures of infection control between October 11th and October 27th. The method of active screening and targeted environmental monitoring was described previously [23]. In brief, for active screening, we collected samples from the nose, throat, groin, and axilla of each patient using sterile cotton swabs (the sputum and stool samples were included in our clinical samples), and a real-time PCR assay was used to screen all samples for *bla*_KPC_ [24]. The first swab of each patient was taken within 48 h upon ICU admission. For targeted environmental monitoring, six environmental sites on the bed sheet were sampled for each KPC-KP positive patient using contact plates (16 cm^2^; Qingdao Classical Biochemical Equipment, Qingdao, China).

### Bacterial identification and antimicrobial susceptibility

Bacterial identification and antibiotic susceptibility testing were initially performed using the VITEK-2 automated system (BioMérieux, France). *Escherichia coli* ATCC 25922 was used as quality control strain for antibiotic susceptibility testing. Susceptibility category was designated according to the Clinical and Laboratory Standards Institute (CLSI) breakpoints [22]. Susceptibility to tigecycline was defined based on the criteria proposed by the European Committee on Antimicrobial Susceptibility Testing-2011 (susceptible, minimum inhibitory concentration (MIC) ≤ 1 μg/ml).

Phenotypic screening for the presence of carbapenemase was performed using the modified Hodge test (MHT). In addition, ESBL production was tested using the Double-Disk Synergy Test (DDST), as recommended by the CLSI [22].

### Determination of carbapenemase genes

We screened carbapenem-resistance genes (*bla*_KPC_, *bla*_NMC_, *bla*_SME_, *bla*_IMI_, *bla*_GES_, *bla*_NDM_, *bla*_IMP_, *bla*_VIM_, *bla*_SPM_, *bla*_GIM_, *bla*_SIM_, *bla*_OXA-48_, *bla*_OXA-51-like_, *bla*_OXA-23-like_, *bla*_OXA-24-like_, *bla*_OXA-58-like_, in all strains as described previously [5, 25, 26]. DNA sequencing was performed on both strands of the PCR amplification products. The results were compared and aligned with reference sequences using the online BLAST database.

### Pulsed-field gel electrophoresis (PFGE)

We used the 1-day, standardized PFGE protocol for *K. pneumoniae* [27]. Cell suspensions were placed in polystyrene tubes (Falcon; 12 × 75 mm), and their optical density was adjusted to 3.8–4.0 using a Densimat photometer (BioMérieux, Marcy l’Etoile, France). *K. pneumoniae* slices were digested using 50 U per slice of *Xba*I (Takara, Dalian, China) for 4 h at 37 °C, and electrophoresis was performed using a CHEF-DRIII system (Bio-Rad Laboratories, Hercules, CA, USA). Electrophoresis was run with a switch time of 6 s to 36 s for 18.5 h, and images were captured using a Gel Doc 2000 system (Bio-Rad) and converted to TIFF files. The TIFF files were analyzed using the BioNumerics version 5.1 software (Applied Maths, Kortrijk, Belgium).

### Multilocus sequence typing (MLST)

MLST with seven genes (*gapA*, *infB*, *mdh*, *pgi*, *phoE*, *rpoB*, and *tonB*) was performed on isolates according to the protocol described on the *K. pneumoniae* MLST website (http://bigsdb.pasteur.fr/). Alleles and STs were assigned using the MLST database (http://bigsdb.pasteur.fr/klebsiella/klebsiella.html). Alleles and STs that had not been previously described were submitted to the curator of the database and were assigned new designations.

### Whole genome sequencing (WGS), detection of single nucleotide polymorphisms (SNPs), and clustering analysis

The initial strains of each case were selected for WGS. Bacterial strains were sequenced using Illumina sequencing by constructing two paired-end (PE) libraries with average insertion lengths of 500 bp and 2000 bp, respectively. Sequences were generated using an Illumina GA IIx (Illumina Inc., San Diego, CA, USA). Raw data was processed in four steps, including removing reads with 5 bp of ambiguous bases, removing reads with 20 bp of low quality (≤ Q20) bases, removing adapter contamination, and removing duplicated reads. Finally, 100× libraries were obtained with clean PE read data. Assembly was performed using SOAPdenovo v1.05 [28].

The whole-genome sequence of *K. pneumoniae* HS11286 (GenBank accession: NC_016845.1) was used as the reference sequence, and clean reads of sequenced isolates were mapped to the reference genome by bowtie 2 software under the default parameters [29]. SNPs were then identified using Samtools [30] and combined together according to the reference. SNPs with low quality (read depth < 5) and those located within 5 bps on the same chromosome were removed to avoid the effect of recombination, as described in our previous studies [31, 32]. The isolates were clustered and a heatmap was generated using the heatmap package in R. The transmission route was then reconstructed based on the emergence of different SNPs in each isolate and the case information, including the onset time of infection and the hospitalization time.

## Results

### Outbreak descriptions

The outbreak occurred in the SICU of Beijing Tongren Hospital, a 1600-bed general tertiary care and university-affiliated teaching hospital in Beijing, China. Beijing Tongren Hospital receives an average of 3500–5000 outpatients and emergency patients per day. SICU has 18 beds.

**Fig. 1:**
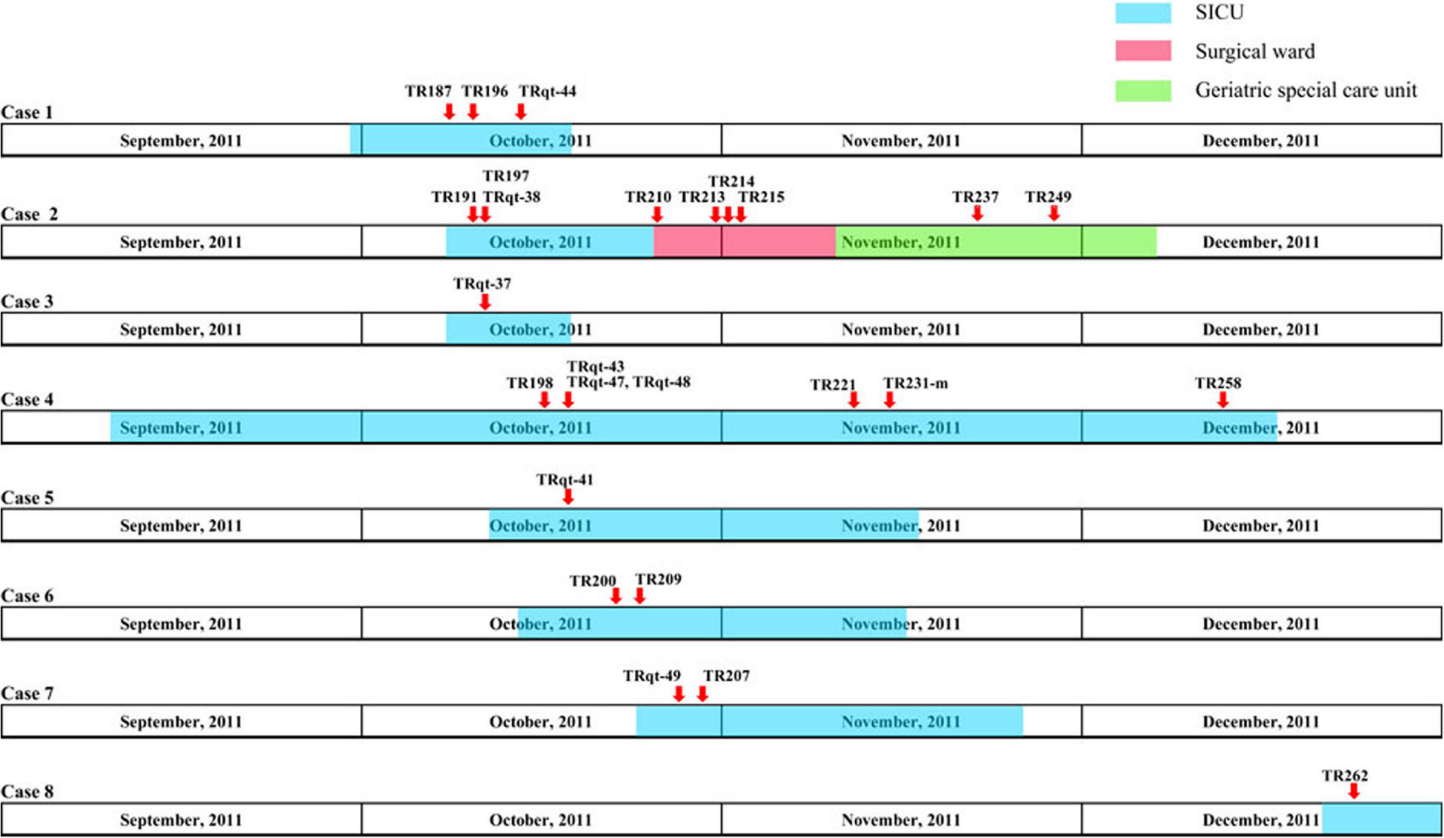
The timeline of patient admission and CRKP isolation. Shadows on the timeline represent the admitting’s duration of the case. Different wards are indicated in different colors. The red arrow indicates the isolation of the strains

The index case of this outbreak was identified on October 8th, 2011. Following identification of second CRKP-infection case in the same room on October 10th, and a CRKP carrier on October 11th, an outbreak was declared. Between October 8 and December 23, a total of 50 patients were admitted into the SICU and eight of them were found to have a CRKP infection or colonization though routine clinical culture and active screening (Table 1). The timeline of patient admission and CRKP isolation is showed in Fig. 1.

**Table 1:** Case descriptions involved in the outbreak of SICU, October to December 2011

Case No.	Time of stay in SICU	Date of the first isolation	Type of specimen	Infection /colonization	Antimicrobial used before^a^	Antimicrobial used after	Outcome
1	30/9/2011–17/10/2011	8/10/2011	sputum	infection	one course of VA	one course of AK	died
2^b^	7/10/2011-14/10/2011	10/10/2011	abdominal drainage fluid	infection	one course of MEM	one course of AK	transfer to other ward
3	8/10/2011–17/10/2011	11/10/2011	nose	colonization	–	–	discharged
4^c^	10/9/2011-16/12/2011	16/10/2011	throat	infection	one course of CAZ and 1 course of PIP/TAZ	two courses of Gn	transfer to other ward
5	14/10/2011–16/11/2011	18/10/2011	nose	colonization	one course of CIP	one course of Ak	discharged
6	16/10/2011–15/11/2011	21/10/2011	urine	infection	one course of CRO	one course of GN	discharged
7	24/10/2011–25/11/2011	27/10/2011	nose	colonization	one course of CRO	one course of CRO	discharged
8	20/12/2011–22/1/2012	23/12/2011	sputum	colonization	–	–	discharged

^a^*VA* Vancomycin, *MEM* meropenem, *CAZ* ceftazidime, *PIP/TAZ* piperacillin/tazobactam, *CIP* ciprofloxacin, *CRO* ceftriaxone, *AK* amikacin, *GN* gentamicin

^b^ Case 2 was transferred to a general surgery ward and ultimately discharged from the hospital

^c^ Case 4 was transferred to a geriatric ward and ultimately discharged from the hospital

Among the eight patients, four were infected by CRKP and other four were CRKP carrier. Of the four infection cases, one died, one was discharged from the SICU, and two were transferred to other wards and ultimately discharged. It should be noted that cases 1 to 7 overlapped with at least three other cases when in the SICU, but case 8 did not overlap with any other case during SICU stay.

CRKP detected twice from the the bed sheets of case 4 during the outbreak. In addition to active screening and targeted environmental monitoring, other stringent infection prevention and control measures were implemented, including contact precautions, strengthen of hand hygiene, environmental cleaning (including changing the bed linen more frequently for each CRKP-positive patient, and immediately stringent terminal sterilization with hydrogen peroxide after they were discharged from the ward or died), and enhanced antimicrobial stewardship were introduced. For the patients transferred to other wards, contact precautions and follow-up screening were employed until they were discharged from our hospital. Since January 22, 2012 (the discharge date of the last patients with CRKP colonization), over a period of 10 months, no further carbapenem-resistant *Enterobacteriaceae* (CRE) were isolated in the SICU.

### Antibiotic susceptibility and characterization of resistance genes

All 26 CRKP isolates showed same results of antibiotic susceptibility test. The MICs of meropenem, imipenem, and ertapenem were ≥ 16 μg/ml, ≥ 16 μg/ml, and ≥ 8 μg/ml, respectively, for all isolates. All isolates were susceptible to amikacin ((MIC ≤2 μg/ml), gentamicin (MIC ≤1 μg/ml), tobramycin (MIC ≤1 μg/ml), and trimethoprim/sulfamethoxazol (MIC ≤1:19 μg/ml), and were resistant to ampicillin (MIC ≥32 μg/ml), ampicillin/sulbactam (MIC ≥32 μg/ml), piperacillin (MIC ≥128 μg/ml), piperacillin/tazobactam (MIC ≥128 μg/ml), cefazolin (MIC ≥64 μg/ml), cefotetan (MIC ≥64 μg/ml), ceftazidime (MIC ≥64 μg/ml), ceftriaxone (MIC ≥64 μg/ml), cefepime (MIC ≥64 μg/ml), aztreonam (MIC ≥64 μg/ml), ciprofloxacin (MIC ≥4 μg/ml), levofloxacin (MIC ≥8 μg/ml), nitrofurantoin (MIC ≥512 μg/ml), and tigecycline (MIC ≥2 μg/ml). All isolates were positive for carbapenemase and ESBL production by the MHT and DDST assays, respectively. We further confirmed the presence and production of carbapenemases and ESBLs by PCR and sequencing. All 21 isolates harbored the *bla*_KPC-2_, *bla*_SHV-1_, and *bla*_CTX-M-15_ genes and tested negative for other antimicrobial resistance genes (*bla*_NMC_, *bla*_SME_, *bla*_IMI_, *bla*_GES_, *bla*_NDM_, *bla*_IMP_, *bla*_VIM_, *bla*_SPM_, *bla*_GIM_, *bla*_SIM_, *bla*_OXA-48_, *bla*_OXA-51-like_, *bla*_OXA-23-like_, *bla*_OXA-24-like_, *bla*_OXA-58-like_, and *bla*_TEM_).

### Molecular subtyping analysis by PFGE and MLST

**Fig. 2:**
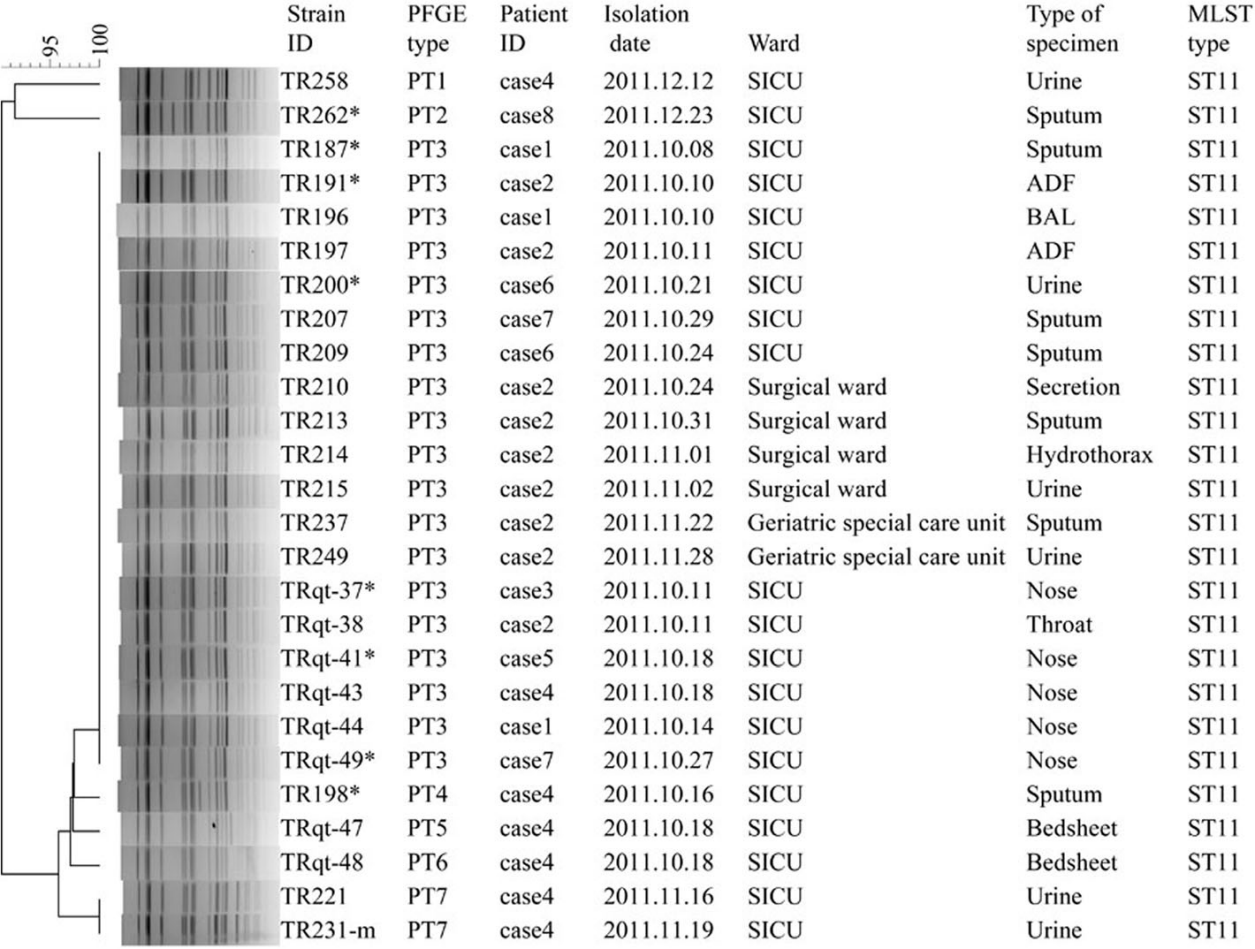
Clustering of the 26 *K. pneumoniae* isolates based on PFGE patterns. The first positive cultures of each case are marked by an asterisk. The information of strain ID, PFGE type, patient ID, isolation date, ward, type of specimen, and MLST type is listed to the left of the patterns. ADF, abdominal drainage fluid; BAL, bronchoalveolar lavage

MLST indicated that all 26 isolates belonged to ST11. However, PFGE showed some diversity (Fig. 2). PFGE divided the 26 isolates into seven different PFGE types (PT1–PT7). The dominant PFGE type (PT3) contained 19 isolates. The other six PFGE types showed one to four bands that were different to the dominant PFGE type (PT3). Except for cases 4 and 8, all the first positive cultures from each affected patients belonged to the dominant PFGE type (PT3). The isolates of case 4 (PT4) and case 8 (PT2) showed one and three bands that were different to those of PT3, respectively. Using the interpretation criteria of PFGE patterns proposed by Tenover et al. [33], the first positive culture of cases 1, 2, 3, 5, 6, 7 were the “same strain” and those of cases 4 and 8 were “closely-related strains”.

Excepting for case 8, all six isolates showing different patterns to the dominant PFGE type were isolated from case 4, including three strains from urine, one from sputum, and two from the bed sheet. The two strains isolated from the bed sheet (TRqt-47 and TRqt-48) of case 4 showed one band that was different to the strain isolated from the nose (TRqt-43) on the same day. It was interesting that with increasing time, the PFGE patterns of the strains from case 4 also changed, especially isolates from December 2011. All the strains isolated in October showed dominant patterns or showed only one band different from the dominant pattern; two strains isolated in November (TR221, TR231-m) showed two bands that were different from dominant pattern and another strain isolated in December (TR258) showed four bands that were different from dominant pattern.

### Comparison of outbreak isolates based on WGS-based SNPs

**Fig. 3:**
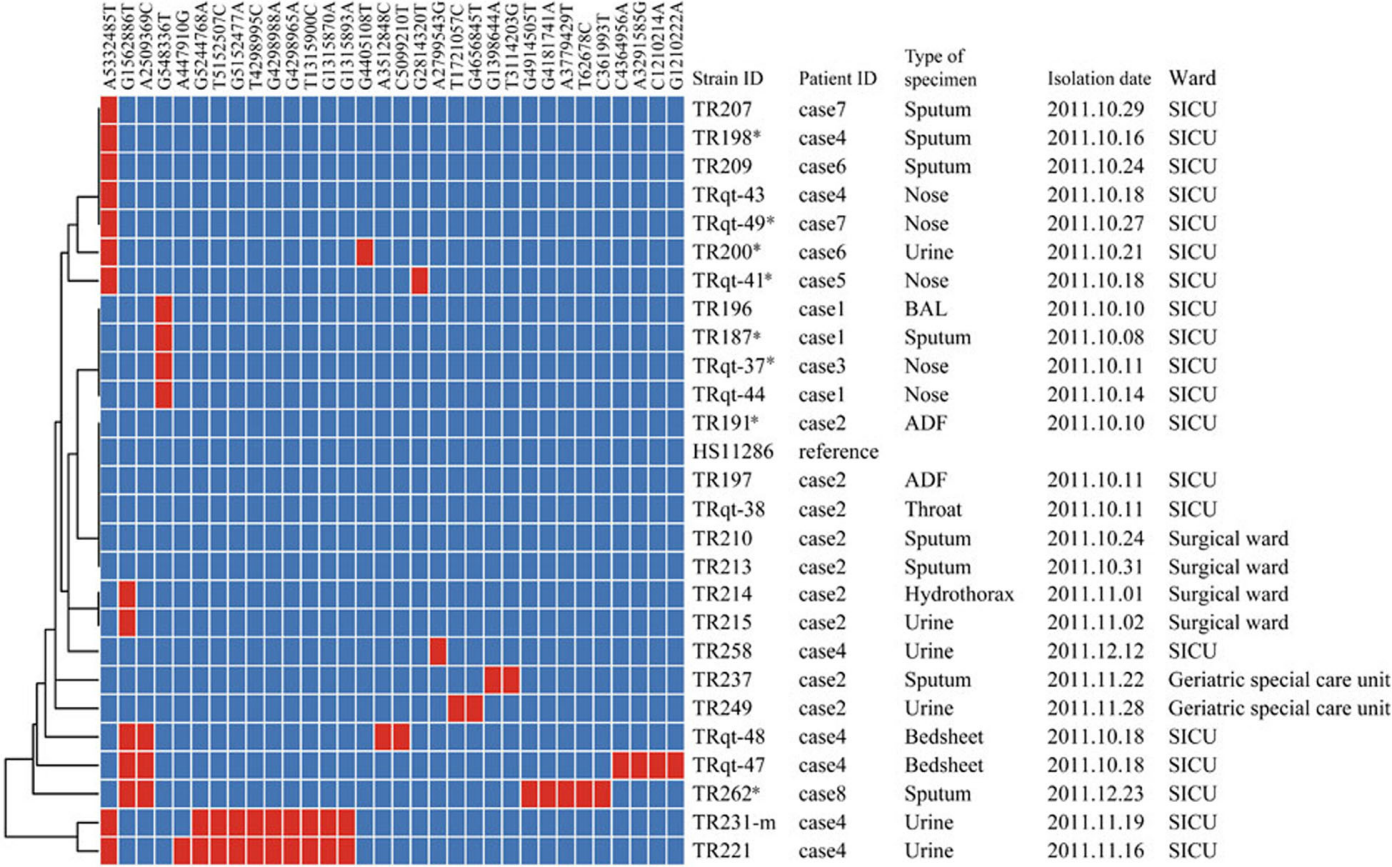
Clustering of the 26 *K. pneumoniae* isolates based on MCG typing. The first positive cultures of each case are marked by an asterisk. The information of strain ID, patient ID, type of specimen, isolation date, and ward are listed to the left of the patterns. ADF, abdominal drainage fluid; BAL, bronchoalveolar lavage

**Fig. 4:**
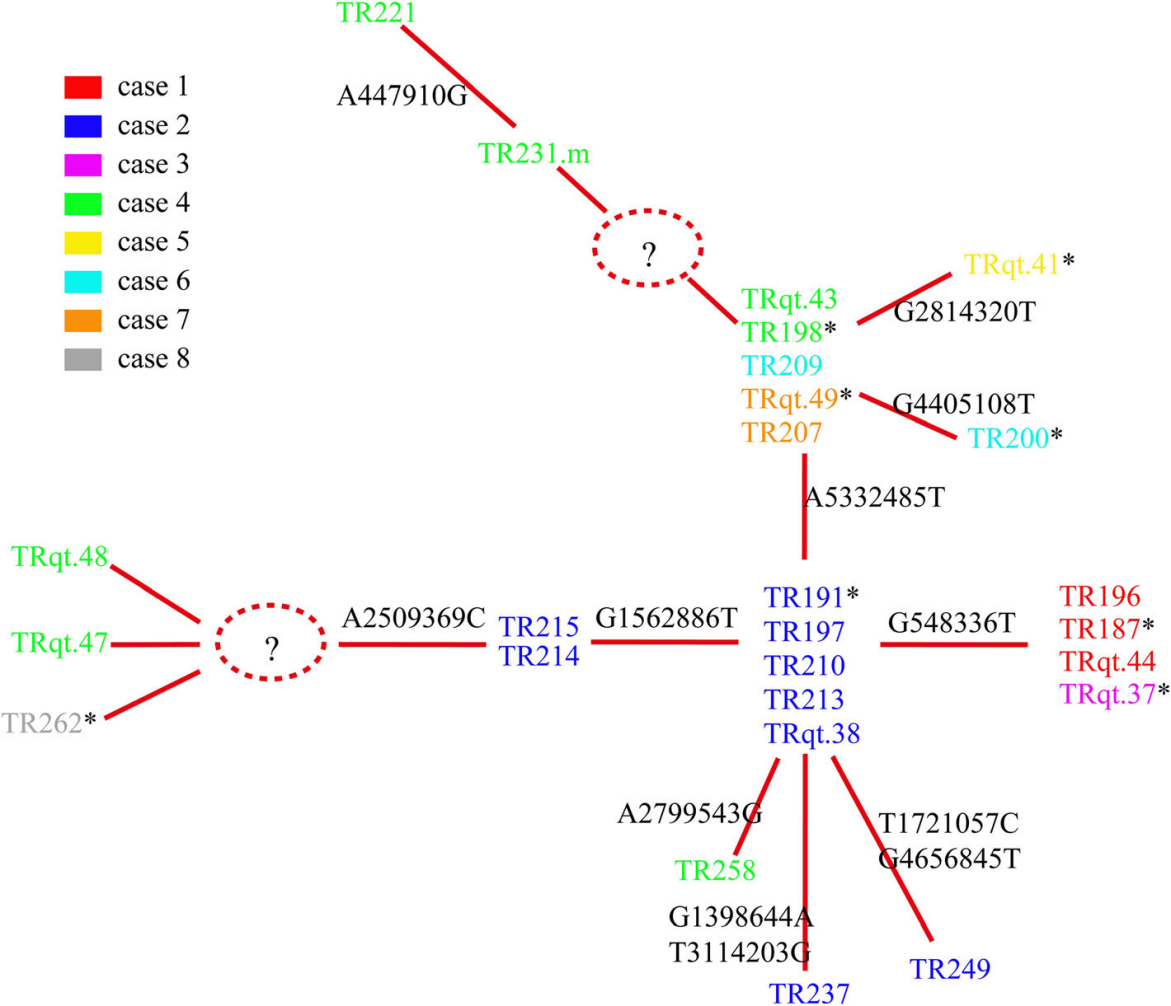
Evolutionary relationships based on MCG typing of 26 CRKP isolates. Isolates from different patients are indicated in different colors. The first positive cultures of each case are marked by an asterisk

We performed WGS and MCG typing of the 26 CRKP isolates, in hope of using the SNPs found in their genomes to determine putative transmission map of this outbreak. Genomic comparisons revealed a total of 32 MCG SNPs among the 26 isolates (Fig. 3). The evolutionary relationships based on MCG typing of the 26 CRKP isolates are outlined and presented in Fig. 4.

Five case 2 strains isolated in October showed the same SNP profile as the reference strain *K. pneumoniae* HS11286. The other case 2 strains isolated in early and late November had one and two SNPs that were different to the reference strain, respectively. All isolates from cases 1 and 3 had only one SNP difference to the reference strain. At the same time, two isolates from case 4, one isolate from case 6, and two isolates from case 7 had the same SNP profile and showed only one SNP difference to the reference strain.

It is worth noting that four case 4 isolates, two from the bed sheet (TRqt-47, TRqt-48) and two from urine (TR221, TR231-m), showed 7–10 SNP differences compared with the initial isolate (TR198) of case 4, and another urine isolate of case 4 (TR258) isolated later had only two SNP differences to TR198, suggesting that the CRKP isolated from case 4 during the study period came from a different source.

**Fig. 5:**
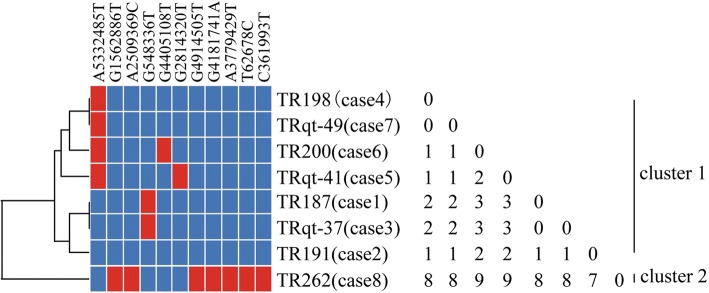
Clustering of the eight first positive CRKP cultures of each case based on MCG typing. The matrix of the SNP differences is presented on the left of the cluster tree

The isolate of case 8 (TR262) showed 6–18 SNP differences with other isolates and had five specific SNPs. To determine whether case 8 is related to the other seven cases, the initial isolates of each case were chosen and analyzed. Grouping the eight initial isolates on the basis of the patterns of shared variants partitioned them into two clusters (Fig. 5). Cluster 1 contained seven isolates of cases 1 to 7, and cluster 2 consisted only of case 8. The pairwise distances within cluster 1 were ≤ 3 SNP differences; in contrast, the distances between cluster 2 and cluster 1 isolates were 8 SNP differences. The within-cluster distance of cluster 1 was much smaller than the between-cluster distance, indicating considerable divergence between the two clusters. The epidemiological information of case 8 indicated that they had not overlapped during their hospital stay with any of the other seven patients; therefore, we deduced that cases 1 to 7 probably shared the same transmission route and that case 8 seemed to be an independent event.

### Inference of most likely transmission route

**Fig. 6:**
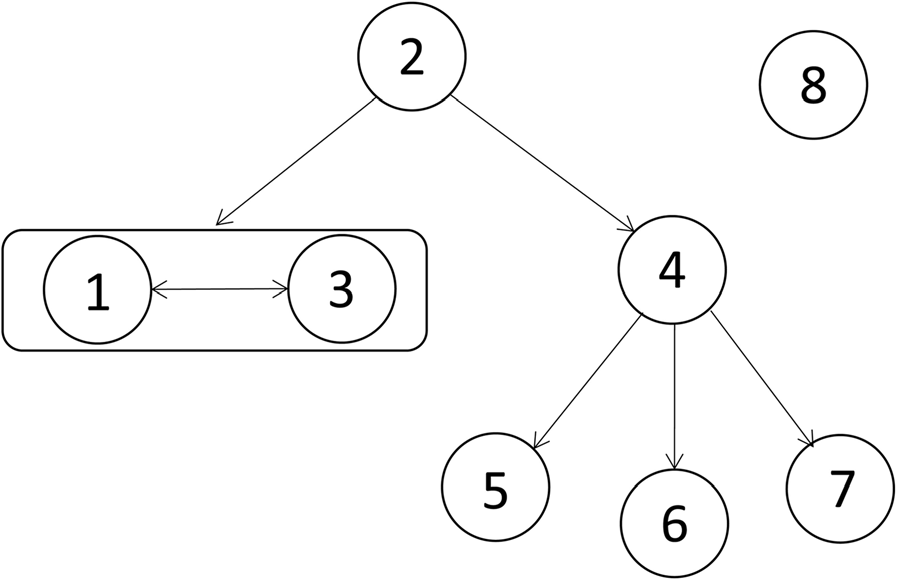
Putative map of CRKP transmission during the outbreak. The transmission map was constructed with genetic and patient trace data. Nodes represent patients, and arrows indicate a transmission event directly from one patient to another

Furthermore, the genomic and epidemiological information were integrated to construct the transmission route. The most likely transmission route is shown in Fig. 6. First, case 2 was suspected of being the source of the outbreak isolates because the CRKP from case 2 showed the same SNP patterns to reference strain *K. pneumoniae* HS11286. This was also supported by the epidemiological information. Case 1 admitted in the SICU on September 30th, 2011, 9 days before the first CRKP was isolated, which suggested that the CRKP of case 1 was acquired in the hospital. Thus Case 1 is unlikely to be the source. Case 3 checked in the SICU on October 8th, 2011, the same day of first CRKP isolation of case 1; however, case 3 checked in the SICU in the afternoon, and the CRKP-positive sample had already been collected from case 1 in the morning, which suggested that the CRKP of case 1 was not transmitted from case 3. Thus, case 2 was the most likely source of the outbreak isolates.

Second, the isolates of cases 1 and 3 showed no SNP differences, suggesting direct transmission between them. However, it could not be confirmed that whether case 2 directly transmitted CRKP to case 1 or case 3.

Third, the case 2’s isolate showed one SNP difference to isolates of cases 4 and 7, but two SNP differences to that of cases 5 and 6, suggesting that CRKP from case 2 was genomically closer to cases 4 and 7. However, case 7’s hospital stay did not overlap with that of case 2 and the date of the first isolation of CRKP from case 7 was significantly later than the other cases; therefore, we deduced the case 7’s isolate was transmitted from case 2 through case 4. The isolates of cases 5 and 6 were transmitted from case 4, as there was only one SNP between isolates cases 5 and 6 and that from case 4, but showed more SNPs differences to that of other cases.

Consistent with the genomic and epidemiological information, the integrated map identified two transmissions starting from case 2 (Fig. 6). The first transmission was directly from case 2 to case 1 or case 3, and a transmission was observed between cases 1 and 3. A second transmission from case 2 was predicted to go through case 4 before being transmitted to cases 5, 6, and 7.

## Discussion

In this study, we described an outbreak caused by CRKP in a SICU of a large university hospital in China. The CRKP isolates belonged to clone ST11, and coproduced carbapenemase (KPC-2) and ESBLs (CTX-M-15 and SHV-1). ST11 is the dominant clone of KPC-producing *K. pneumoniae* in China and has also been reported sporadically in the rest of the world, including other regions of Asia [34, 35], America [36], and Europe [37–39]. KPC is the most common carbapenemase in *K. pneumoniae* and most of the KPC type in China is KPC-2 [40]. Similarly, CTX-M-15 and SHV-1 are the main types of CTX-M-type and SHV-type in China [41]. The coexistence of *bla*_KPC-2_ with *bla*_CTX-M-15_ type genes in *K. pneumoniae* was previously reported in Bulgaria [42], Brazil [43], and China [44]. In a recent study, CRKP strains co-harboring the *bla*_KPC-2_, *bla*_CTX-M-15_, and *bla*_SHV_ genes were found in several STs including ST11; however, only sporadic strains were reported in that study [44]. As far as we know, this is the first study to report an outbreak caused by CRKP co-harboring the *bla*_KPC-2_, *bla*_CTX-M-15_, and *bla*_SHV-1_ genes, which suggests that attention should be paid to the *K. pneumoniae* isolates coproducing epidemic carbapenemases and ESBLs, especially the outbreak strains described here belonging to the epidemic ST11 clone.

Given the easy of transfer and acquisition of carbapenemase and ESBLs genes, measures must be implemented to control the outbreak and avoid nosocomial transmission [2, 45]. Agodi et al. reported that cleaning and disinfection of the ICU, segregation of affected patients, barrier nursing, and strict compliance with hand hygiene procedures led to containment of an outbreak of KPC-producing *K. pneumoniae* [46]. In our study, we applied active screening and targeted environmental monitoring to combat the secondary transmission of imported KPC clones in the SICU. Except for the two index cases, we found that six patients acquired this pathogen during their hospital stay, two of them with urinary tract infections. Some reports stated that the rectum was the most sensitive sampling site for universal screening of CRE [47]. However, during the period of our study, the nose, throat, and sputum were also sensitive sites to detect CRE, which might suggest, indirectly, the important role of the respiratory tract in dissemination during the outbreak. Therefore, it is important to apply active screening with nose, throat, and sputum sampling to detect hospital-acquired cases early during an outbreak.

Additionally, the genetic relatedness between the strains judged by the interpretation criteria proposed by Tenover et al. [33], we drew two additional inferences concerning the strains from case 4. First, with increasing time, the PFGE patterns of strains from case 4 changed, suggesting that genome mutations occurred in the bacteria in vivo for case 4, which was also proved by whole genome sequencing and comparison. Second, the two strains from the bed linen of case 4 were isolated from the same patient at the same time. However the PFGE patterns of the two strains were different. This result may be explained if the two isolates contaminated the bed linen at different times.

Using PFGE, the strain isolated from case 8 was a “closely-related strain” to the other strains. However, case 8 showed weak epidemiological relevance to the other cases, because this case did not overlap with any other case during their SICU stay. So we could not judge the relationship between case 8 and the other cases. We further used WGS-SNP analysis to study the population structure of CRKP isolates from eight cases to reveal the relationships among them. WGS-SNP divided the isolate from case 8 far from the strains of other cases. By combination of the epidemiological information and molecular results, we deduced that (i) cases 1 to 7 were on the same transmission route and that case 8 was an independent event; (ii) case 2, but not case 1 (the index patient), was the source of CRKP in this outbreak; (iii) there were two transmissions starting from case 2.

## Conclusions

Though this study is a retrospective study and thus the results of the WGS could not be used to control the nosocomial transmission. However, our data clearly showed that WGS and MCG typing could reveal the details of transmission within a CRKP nosocomial outbreak. In the future, real-time genomic sequencing and analysis of an outbreak should be carried out and the findings could be used to control outbreaks.

## Abbreviations

CLSI: Clinical and Laboratory Standards Institute
CRE: carbapenem-resistant *Enterobacteriaceae*
CRKP: Carbapenem-resistant *K. pneumoniae*
DDST: Double-Disk Synergy Test
ESBL: extended-spectrum β-lactamase
KPC: *K. pneumoniae* carbapenemase
MHT: modified Hodge test
MIC: minimum inhibitory concentration
MLST: Multilocus sequence typing
PFGE: Pulsed-field gel electrophoresis
SICU: surgical intensive care unit
SNP: detection of single nucleotide polymorphism
ST: sequence type
WGS: Whole genome sequencing
